# Addressing the elephant in the room, therapeutic resistance in non-small cell lung cancer, with epigenetic therapies

**DOI:** 10.18632/oncotarget.8205

**Published:** 2016-03-19

**Authors:** Corey A. Carter, Karen Zeman, Regina M. Day, Patrick Richard, Arnold Oronsky, Neil Oronsky, Michelle Lybeck, Jan Scicinski, Bryan Oronsky

**Affiliations:** ^1^ Walter Reed National Military Medical Center, Bethesda, MD, USA; ^2^ National Naval Medical Center, Bethesda, MD, USA; ^3^ Uniformed Services University of The Health Sciences, Bethesda, MD, USA; ^4^ InterWest Partners, Menlo Park, CA, USA; ^5^ CFLS Data, Mountain View, CA, USA; ^6^ EpicentRx, Inc, Mountain View, CA, USA

**Keywords:** non small cell lung cancer, oncology, epigenetics, resistance

## Abstract

Like Chinese boxes nesting inside each other, the classification of non-small cell lung cancer (NSCLC) is subdivided into smaller and smaller subtypes on the basis of histological and molecular attributes. The latter characterizes NSCLC by its molecular alterations and the identification of inhibitors that target these cancer-specific “driver” mutations. Despite the initial promise of precision-guided therapies to inhibit a finer and finer array of molecular subcategories, despite even the curative potential of immunotherapeutic checkpoint blockade, in particular, casualties still abound and true clinical success stories are few and far between; the ever-present, if sometimes unmentioned, “elephant in the room”, is the acquisition of resistance, which, sooner or later, rears its ugly head to undermine treatment success and shorten survival. Emerging data suggests that epigenetic therapies are able to reprogram the aberrant tumor-associated epigenome and ‘tame the beast of resistance’, thereby prolonging survival. This article reviews the role of epigenetic dysregulation in NSCLC, explores PFS2 as a possible surrogate endpoint, briefly mentions possible biomarkers and highlights combinatorial treatment epigenetic strategies to “prime” tumors and reverse resistance.

## INTRODUCTION

The success stories of childhood ALL, testicular cancer, and Hodgkins’ disease notwithstanding, malignancies that were once universally fatal, but are now generally curable, the progress on other oncology fronts has been, at best, lamentably slow or, at worst, virtually nonexistent with the prospects for long-term survival generally measuring in months rather than years [1].

Lung cancer, in particular, dominates as the leading cause of death among men and women in North America, an unenviable distinction that the three other most prevalent types of cancers, colon, breast, prostate, fortunately do not come close to sharing [2]. It is expected that there will be approximately 221,200 new cases of lung cancer in 2015, accounting for about 13% of all cancer diagnoses [3, 4]. As the predominant form of lung cancer, accounting for 80-85% of the disease, [5, 6, 7, 8, 9]. Non Small Cell Lung Cancer (NSCLC) is the focus of this review.

An estimated 172,016 patients were diagnosed with NSCLC in 2009 [2]. For lung cancer patients who will be diagnosed under current conditions, approximately 65% present with locally advanced or metastatic disease [10], and more than 55% will present with advanced stage disease (stage IV or stage IIIB) that is not amenable to curative treatment. Of the remaining 45% that are treated with curative intent only 20% of these will undergo surgery while the remaining will be treated with definitive chemoradiation. More than half of these patients will relapse and eventually succumb to their disease [2, 11]. There is no current consensus regarding the optimal treatment strategy for NSCLC [12, 13, 14]. Current therapeutic options in NSCLC, reviewed briefly below, are plagued by the emergence of resistance and cross-resistance[15, 16], practically a *fait accompli* and an inevitable consequence of exposure to treatment, highlighting the urgent need for strategies that are able to circumvent and overcome it [17, 18, 19, 20].

The philosopher, George Santayana, famously wrote: “Those who cannot remember the past are condemned to repeat it.”[21] A Santayana-like guiding principle in NSCLC eschews the *déjà vu*, precluding repeated exposures to past chemotherapies on the premise that the risk of toxicity outweighs the potential for clinical benefit. Therefore, in general, at the moment resistance occurs, a new line of therapy is proactively introduced, progressively narrowing options as patients relentlessly progress forward, never backward, through the treatment funnel, 1^st^ line, 2^nd^ line, and 3^rd^ line treatments, on the way to hospice and death.

In first line a number of platinum-based doublet compounds represent the current standard of care for squamous and non-squamous mutation negative or unknown status [22, 23, 24]. For EGFR mutation positive non-squamous, the first line therapy is the tyrosine kinase inhibitor, gefitinib [25, 26, 27, 28, 29]. The median survival of all patients with advanced NSCLC is 10-12 months [2]. The fraction of patients who survive one year after diagnosis has only increased slightly over the past decade. Treating patients with more than 4 cycles of the same chemotherapy, or adding a third chemotherapy agent to the platinum-based doublet has not improved overall survival [30]. Non-platinum-based regimens have been evaluated in several phase II and phase III clinical trials, showing survival rates similar to platinum based treatments [5]. The EORTC- 08975 randomized 483 patients to three arms—two cisplatin based regimens and one non-cisplatin based (paclitaxel/gemcitabine). The response rates were similar (27.7% to 36.6%, respectively) as was the median survival (between 6.7 months to 8.9 months), but progression free survival was inferior in the non-platinum based regimen. Other studies have reported somewhat worse results with non-platinum combinations; therefore platinum is still considered a preferred agent for combination chemotherapy in fit patients with advanced NSCLC [31, 32, 33, 34, 35].

Second line treatment for recurrent or progressive disease includes treatment with the chemotherapy agents docetaxel and pemetrexed, or treatment with an oral EGFR antagonist, erlotinib [36], in the case of mutation negative non-squamous NSCLC or erlotinib or docetaxel for squamous NSCLC [37]. Docetaxel was approved for second line treatment in 2000 after a randomized trial demonstrated that docetaxel at 75 mg/m^2^ given every 3 weeks offered a clinically meaningful benefit to patients with advanced NSCLC whose disease had relapsed or progressed after platinum-based chemotherapy with a response rate of approximately 7%. Pemetrexed was approved as a second line treatment based on a non-inferiority study as compared to docetaxel [38]. Treatment with pemetrexed resulted in clinically equivalent efficacy outcomes, but with significantly fewer side effects compared with docetaxel in the second-line treatment of patients with advanced NSCLC. With these findings pemetrexed is increasingly used in the second line setting, and recent clinical trials have extended its use in first line setting for the treatment of patients with non-squamous cell carcinoma NSCLC [39]. A phase III trial compared pemetrexed/cisplatin to gemcitabine/cisplatin. This trial has demonstrated an improvement in overall survival over the combination cisplatin/gemcitabine in patients with adenocarcinoma NSCLC (12.6 vs. 10.9 months) and in large cell NSCLC (10.4 vs. 6.7 months) [40].

The decision to use pemetrexed depends on the histology of NSCLC. A recent review of two large pemetrexed based trials concluded that although pemetrexed is well tolerated in squamous cell histology patients, pemetrexed-based regimens result in a shorter overall survival than non-pemetrexed-based regimens in treatment of recurrent squamous cell NSCLC [41]. With the increased frequency of pemetrexed use in the first-line setting for adenocarcinoma and its limitation to non-squamous cell histology, the need for improved second line agents is increasingly evident [42]. Currently, patients with advanced NSCLC who have progressed after 2^nd^-line treatment have limited options [43]. Retrospective analysis using chemotherapy for 3^rd^ line treatment demonstrates response rates of only 2% and median survival of 4 months [44].

Oral agents gefitinib and erlotinib are small molecule inhibitors of the EGFR tyrosine kinase activity, which have demonstrated clinical benefit after failure of first line chemotherapy [45, 46]. Erlotinib was approved in 2004 for the treatment of patients with locally advanced or metastatic NSCLC after the failure of one or two prior chemotherapy regimens with a single agent response rate of 8.3 % and a median overall survival of 6.3 months [47]. To date it remains the only approved third line therapy for NSCLC. The seductively simple appeal of molecularly-targeted agents like erlotinib, which involves turning off addicted oncogenic targets, is belied by broad toxicity profiles and the rapid evolution of mutant inhibitor-resistant kinases [48], lurking in the molecular background, resulting in regrowth of resistant tumors and the erosion of any survival gains.

**Figure 1:**
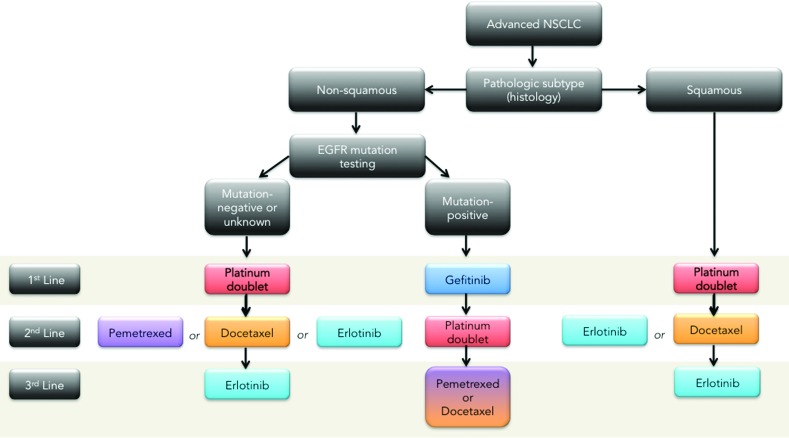
NSCLC therapeutic management algorithm (maintenance therapy not included)

This overview of therapeutic management algorithm (Figure 1) underscores the direness and the urgency of the situation in NSCLC where options are limited and the rapid dynamics of resistance have frustrated efforts to prolong the 5-year survival rate, which, rather depressingly, has plateaued at 15% in North America for over three decades [49, 50, 51, 52] ; even checkpoint inhibitors, which “really work” systemically to control the disease, are susceptible to the development of resistance [53, 54, 55, 56, 57, 58, 59].

Cancer cells are advantaged in terms of mutability and evolvability, readily adapting in the face of environmental change to avoid eradication. In common with other opportunistic pathogens i.e. viruses, bacteria, protozoa, fungi, and parasites it is distinctly possible that no matter how active the regimen cancer cells will always find a way to overcome its therapeutic effects. Fortunately, a resistance reset button may exist in the form of epigenetic agents, which, despite apparently negligible activity as single agents in NSCLC, may in concurrent and sequential combination improve the activity of standard chemotherapy agents through alteration of the epigenome [60, 61].

The cancer epigenome is characterized by global changes in DNA methylation and chromatin, such as the hypermethylation of promoter-associated CpG-islands [62]. These aberrations, which disrupt normal gene expression, are an adaptive and essential tactic [63] to provide the tumor with greater agility/phenotypic plasticity in an ever-changing metastatic microenvironment, thereby maximizing fitness and conferring a selective growth advantage to the most resistant cellular subtypes [64]. Two main processes regulate the accessibility of transcription machinery to bind to specific DNA sequences: acetylation of histone proteins responsible for chromatin structure and the methylation of CpG islands on DNA strands. Epigenetic therapies like DNA methyltransferase or histone deacetylase inhibitors, which reactivate epigenetically silenced genes and cellular pathways, result in phenotypic alterations that potentially revert the malignant cell population to a more normal state and, in this way, render it more susceptible to anti-tumor treatment [65, 66]. Despite initial promise in hematologic malignancies, epigenetic agents have not shown significant efficacy as monotherapy against solid tumors. Recent trials showed that epigenetic agents exert favorable modifier effects when combined with chemotherapy, hormonal therapy, or other epigenetic agents. This article reviews the role of epigenetic dysregulation in NSCLC, explores PFS2 as a possible surrogate endpoint, briefly mentions possible biomarkers and highlights combinatorial treatment epigenetic strategies to “prime” tumors and reverse resistance.

## THE ROLE OF EPIGENETIC AGENTS IN CANCER

The term “epigenetics”, which literally means “above” or “on top of” genetics, currently a “hot topic” in oncology, broadly encompasses alterations or modifications of DNA that modulate gene expression or cellular phenotype but do not affect the primary DNA sequence [67, 68]. As an alternative to genetic mutation, aberrant epigenetic regulation, one of the earliest hallmarks of oncogenesis [69], involves CpG dinucleotide hypermethylation in the promoter regions of tumor suppressor genes and loss of acetylation, resulting in transcriptional repression and a dramatic shift in phenotype, all of which facilitates the adaptation of the cancer cell to its environment [70].

So far, the FDA has approved four epigenetic agents: the methylation inhibitors, decitabine and 5-azacytidine for the treatment of high-risk myelodysplastic syndrome, and the histone deacetylase inhibitors (HDACs), vorinostat or SAHA and romidepsin for the treatment of relapsed or refractory cutaneous T-cell lymphoma [71, 72, 73]. Unlike the precision of molecularly targeted agents, epigenetic inhibitors impact a wide range of targets including tumor suppression from genes like p53 and BRCA1, DNA repair, metabolism and multi-drug resistance due to a broad-spectrum modulation of transcription [74]. In contrast to the heterogeneity of genetic defects in cancer where the range and the reach of mutations greatly exceeds the therapeutic grasp, epigenetic mechanisms are not only accessible and, hence, “druggable” but also reversible, thus the reason for their clinical appeal [75]. Crucially, through this broad transcriptional reprogramming, which results in the altered expression of numerous genes including those for resistance, epigenetic inhibitors may “prepare the ground” and “prime” the tumor to respond more robustly than it otherwise would to additional therapies.

## PFS2 AS A POTENTIAL ENDPOINT FOR EPIGENETIC AGENTS

Studies using epigenetic agents as monotherapies have failed to document a single anti-cancer response, and even in combination therapies with epigenetic agents the response rate is only 6%, yet improved overall survival (OS) trends have been observed and clinical benefit is apparent [76]. The disconnect between response rate and OS with epigenetic agents may be related to the inadequacy of progression free survival (PFS) as an endpoint to measure efficacy, since PFS may ‘miss the forest for the trees’, potentially leading to false-negative conclusions. Epigenetic agents reverse gene silencing, which may lead to p53 activation, rendering tumors cells more susceptible to p53-induced growth arrest, differentiation, and senescence, rather than outright cell death, which challenges the determination and interpretation of clinical benefit [77]. A more relevant endpoint to gauge efficacy in this class of agents, especially in the setting of sequential therapy, when epigenetic inhibitors are used in a “priming” strategy, may be Progression Free Survival 2 or PFS2, an endpoint recently validated by the EMA to measure “time from randomization to objective tumor progression on next-line treatment or death from any cause. In some cases, time on next-line therapy may be used as proxy for PFS.” [78]

**Figure 2:**
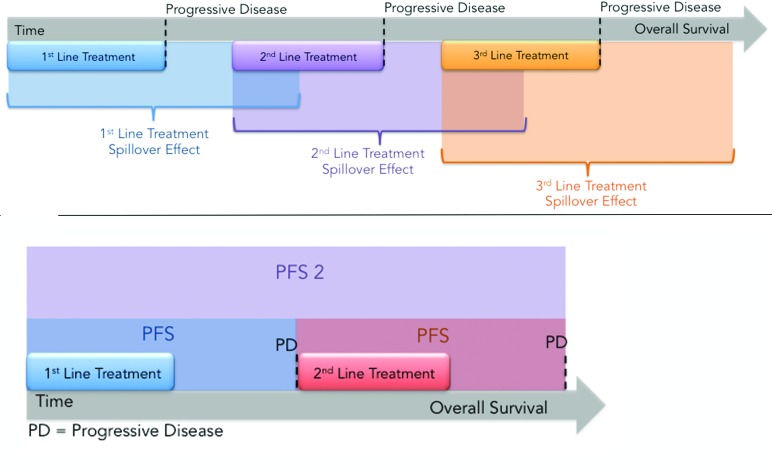
**A.** Treatment Spillover Effect. The effects of each regimen are not confined to the individual line of treatment but spillover and influence subsequent lines of treatment either positively in the case of sensitizing agents or negatively in the case of agents lead to lingering toxicity or that select for resistant clones. PFS does not capture the “spillover effect” while PFS2 does. **B.**Illustration of PFS2, which measures the time from randomization to the time of progression on next line therapy.

What PFS2 measures (or attempts to measure) is therapeutic action-at-a-distance: in other words the effect of the first drug, in this case an epigenetic agent, on subsequent therapies [79]. By analogy with action-at-distance in physics, where gravity, electricity and magnetism transmit an unseen “force field” that acts on other bodies or particles, therapeutic action-at-distance (TAAD) or ‘treatment spillover’ refers to the mutual influence that different agents or regimens separated in time have on each other's activity (Figure 2A).

Even though its basic premise is that present and future events are inseparably intertwined, therapeutic action-at-a-distance is less esoteric and “spooky” (as Einstein called quantum entanglement [80]) than its counterpart in physics: through Newton's 3^rd^ law, the action of chemotherapy elicits a reaction, a pleiotropic adaptive response affecting DNA replication and repair, drug sensitivity, and P-glycoprotein-mediated (Pgp, MDR1, ABCB1) drug efflux that renders the tumor either more sensitive or more resistant to additional therapies. Stated differently, agents can induce or reduce resistance in each other. In either case, but especially with regard to epigenetic inhibitors, PFS2 may be a better and more reliable indicator for prediction of overall survival than PFS because it reflects the causal influence of the present therapy on future therapies. The experimental agent, RRx-001, discussed below, may be the first epigenetic inhibitor compound to test PFS2 as a primary surrogate for overall survival.

## EPIGENETICS IN NSCLC

### DNA methylation

Epigenetic alterations, which include DNA methylation and histone deacetylation, provide a gene silencing mechanism, which underlies progression and therapeutic resistance in NSCLC. With regard to the former, hypermethylation of cytosine in CpG islands by DNA methyltransferases (DNMTs) in the promoter region of tumor suppressor genes leads to their repression and potentiates tumorigenic activity *via* disruption of multiple cellular processes including the cell cycle, DNA repair, and apoptosis [81, 60].

### Histone deacetylation

**Figure 3:**
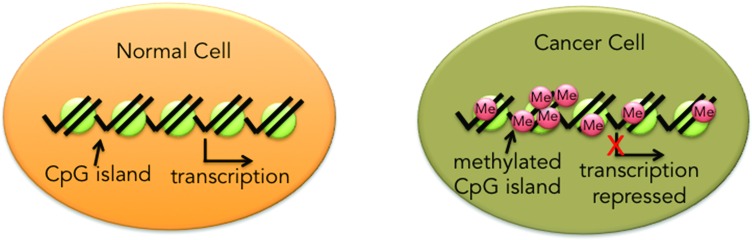
The majority of CpG islands in normal tissue are unmethylated In contrast, CpG island hypermethylation has been described in almost every tumor type and is an important mechanism for transcriptional silencing especially of tumor suppressor genes.

The phenotype of a cell is determined by the pattern of gene expression; that is through the differential transcription of the overall genotype. Chromatin remodeling is one way to alter gene expression. Chromatin is a complex of DNA, histones and non-histone proteins that is organized within the cell's nucleus[82]. Histones are small, positively charged proteins termed H1, H2A, H2B, H3, and H4 that bind with negatively charged DNA and together form repeating subunits called nucleosomes[83]. Histones are subject to a diverse set of modifications, including reversible acetylation, which mediates histone-DNA interactions through electrostatic mechanisms and thereby enhances or blocks the access of transcription factors to specific DNA promoter regions [84]. Deacetylated histones lead to transcriptional inactivation (gene repression) whereas acetylated histones are linked to transcriptional activation (increased gene expression) [85]. Histone deacetylase (HDAC) enzyme activity leads to a tightly packed, less accessible, and less actively transcribed state of DNA, while conversely histone acetylation, controlled by histone acetyltransferases, favors an open, more loosely packed state, leading to DNA transcriptional activation [86]. HDAC overexpression and overactivity has been documented in many cancers including NSCLC [87] (Figure 3).

Histone N-terminal tails are central to the processes that modulate chromatin structure and function, which, in turn, influences the accessibility and activity of the transcription machinery. Post-translational modifications (PTM) of four lysine residues, K4, K9, K27 and K36, in the N-terminal tails of histone H3 are a key element in the epigenetic regulation of gene expression. In particular, trimethylation of histone H3 at lysine 27 (H3K27me3) is associated with transcriptional repression. The Polycomb repressive complex, PRC2, mediates this H3K27 methylation [88]. The catalytic subunit of PRC2, EZH2, implicated in the proliferation and progression of NSCLC [89], is thus a potential target for epigenetic inhibition.

### DNA methylation as a prognostic marker in NSCLC

As a new paradigm in the treatment of cancer, epigenetic priming may benefit from the discovery and validation of actionable biomarkers to identify patients likely (or unlikely) to respond. Several studies [60] have suggested that the presence of DNA hypermethylation in NSCLC might be associated with progression, recurrence, and long-term survival [90]. In one of these clinical trials, a nested case-control study of stage I NSCLC patients with and without recurrent disease, promoter methylation of the cyclin-dependent kinase inhibitor 2A gene, p16, and the H-cadherin gene, CDH13 was associated with recurrent cancer when the primary tumor and resected lymph nodes were evaluated by a multiplex methylation-specific PCR assay [91].

## CLINICAL TRIALS WITH EPIGENETIC AGENTS

### Hydralazine and valproate

As one of the earliest indications that epigenetic therapies could be used to overcome therapeutic resistance, Candeleria *et al*. added hydralazine, a histone deacetylase inhibitor, and valproate, a DNA methyltransferase inhibitor, to the regimen of 15 patients with different tumor types that were progressing on standard chemotherapy in an open-label 2007 Phase II study. Clinical benefit was observed in 12/15 (80%) patients: four partial responses and eight stable diseases. At the same time, quantification of peripheral blood DNA demonstrated reduction in global DNA methylation and histone deacetylase activity [92].

### 5-azacitidine and entinostat

Because monotherapy with DNMT and HDAC inhibitors in solid tumors have demonstrated little clinical activity (with the caveat that the term “clinical activity” may be widely misunderstood and misused with these agents, given their downstream effect on drug sensitivity) combination epigenetic therapy, a preferred strategy to overcome potential mechanisms of resistance, has been tested in clinical trials [93]. In a Phase I/II combination clinical trial of low dose 5-azacitidine (40 mg/m^2^ day 1-6 and day 8) to reduce toxicity, and entinostat (7 mg orally on day 3 and 10 on a 28-day cycle) in heavily pretreated advanced, refractory non-small cell lung cancer (NSCLC), the objective clinical response rate was 4% (2/45), comprising a complete response that lasted for 14 months and a partial response that lasted 8 months. [94] Moreover, 10 additional patients had stable disease that was maintained for 12 weeks. Of particular interest, the authors identified a unique signature of activity in 25% of these refractory patients where response was deferred to the immediate next line of post-study therapy (these therapies included chemotherapy and also immune checkpoint inhibitors [60]), which is compatible with a priming effect on subsequent treatments [94].

### RRx-001

These priming effects have also been observed with RRx-001, a systemically non-toxic dual inhibitor of HDACs and DNA methyltransferases, where so far in a randomized Phase II trial with regorafenib 7/10 colorectal cancer (CRC) patients have been resensitized to previously failed irinotecan for 4 months or longer post-RRx-001 progression, which suggests that RRx-001 epigenetically disrupts multiple cellular processes including chemoresistance, possibly through the de-repression of tumor suppressor genes like p53 [95, 96]. On the basis of these resensitization results and promising preclinical data in pulmonary models [97], a phase II clinical trial acronymed TRIPLE THREAT will open at Walter Reed, in the second-line advanced NSCLC setting as well as third-line small cell lung cancer and second line extrapulmonary neuroendocrine tumors (hence the name TRIPLE THREAT) to examine the role of initial epigenetic therapy with RRx-001 alone until progression followed by rechallenge with platinum doublets to demonstrate resensitization. The primary endpoint is the Overall Response Rate after 6 weeks of platinum-based therapy. However, PFS2 is under consideration as a primary endpoint in the context of a Phase III trial, if the resensitization strategy is successful. In this study, protein hyperacetylation pre- and post-therapy will be assessed in peripheral blood mononuclear cells (PBMC) using multiparameter flow cytometry.

### 5-azacitidine and entinostat followed by nivolumab

In a randomized recently initiated phase II study [98] for second- or third-line advanced NSCLC that examines priming to immune checkpoint inhibitors, patients receive initial epigenetic therapy with 5-azacytidine/etinostat or azacytidine alone for four cycles followed by the antiprogrammed death-1 antibody, nivolumab [60]. According to Forde, Brahmer and Kelly (2014), 5-azacitidine may enhance PD-1 ligand (PD-L1) expression in NSCLC cell lines, suggesting that pretreatment with epigenetic therapy may increase efficacy of anti-PD-1 therapy, which served as the rationale for this trial. Several epigenetic markers will be evaluated in blood and tissue, including candidate promoter methylation markers such as APC, HCAD, p16, RASSF1A, GATA4, and actin to correlate efficacy with biomarker status. [60]According to unconfirmed reports, the initial data are very encouraging.

## DISCUSSION AND CONCLUSIONS

Arguably, the most daunting and omnipresent challenge in oncology is resistance i.e. insensitivity or decreased sensitivity to treatment, which almost always emerges to make its presence felt no matter how initially effective the therapy, recalling the phrase the “elephant in the room”.

Resistance not only renders the current therapy ineffective but also compromises future treatment options through the stepwise accumulation of genetic and epigenetic changes that lead to cross-resistance [99]. Despite a wider armamentarium of off-the-shelf options, including the immune checkpoint inhibitors that have raised expectations with the tantalizing promise of a cure in a percentage of patients, cancer cells, more often than not, manage to escape the consequences of the drug effect, whatever the initial response, and compromise the treatment plan. Unfortunately, resistance is likely here to stay and, in the absence of a cure, tumors will tend eventually to regrow and progress.

One potential solution to this problem is treatment with epigenetic agents, which, with their lower toxicity profiles and ability to attenuate resistance, have the potential, like checkpoint inhibitors, to revolutionize the treatment landscape in NSCLC. Nevertheless, their use as therapeutic “helpers”, “adjuvants”, or “primers” requires patience, a commodity that is generally in short supply in oncology, and a strategic long-view since activity is dependent on the presence of a second or subsequent agent or regimen and may take time to manifest. This tactical priming paradigm suggests that PFS2, the EMA-validated endpoint, is a more appropriate surrogate for overall survival than PFS, since it measures time from randomization to progression on the next-line therapy. In general, as a surrogate, PFS may give a false or misleading signal because, whatever the immediate effect of Therapy A on the tumor, it does not take into account the contribution of Therapy A on resistance mechanisms, which, in turn, influences the response to Therapy B and, by extension, overall survival.

In chess, players may make a move that, on its own, is not beneficial insofar as its only purpose is to set up other subsequent moves. Similarly, the use of epigenetic agents is a chess-like positional play to prime the tumor and pave the way for the ‘next move’, a strategy under intensive investigation in the RRx-001 TRIPLE THREAT trial and checkpoint inhibitor nivolumab trial that sequences cytotoxic and immunologic therapies, respectively, after epigenetic inhibition.

Early encouraging data from these studies in support of the priming hypothesis is fueling enthusiasm for this rapidly evolving epigenetic field, and a full data set is eagerly awaited. The incorporation of biomarkers in these trials could potentially identify responders from non-responders to treatment. In the long term, these biomarkers, if validated, may lead to the personalization of epigenetic therapies (i.e., matching the right patients with the right therapies at the right time). In the meantime, however, the elephant of resistance is still in the room. Even in the absence of definitive proof from clinical trials, it seems like an opportune time to start addressing this gigantic problem with epigenetic agents.
